# *kboolnet*: a toolkit for the verification, validation, and visualization of reaction-contingency (*rxncon*) models

**DOI:** 10.1186/s12859-023-05329-6

**Published:** 2023-06-12

**Authors:** Willow Carretero Chavez, Marcus Krantz, Edda Klipp, Irina Kufareva

**Affiliations:** 1 Skaggs School of Pharmacy and Pharmaceutical Sciences, University of California San Diego, 9500 Gilman Dr, La Jolla, CA 92093 USA; 2 Present Address: Massachusetts Institute of Technology, 77 Massachusetts Ave, Cambridge, MA 02139 USA; 3 Theoretical Biophysics, Humboldt-Universität zu Berlin, Invalidenstr. 42, 10115 Berlin, Germany; 4 Present Address: School of Medical Sciences and Inflammatory Response and Infection Susceptibility Centre (iRiSC), Faculty of Medicine and Health, Örebro University, Örebro, Sweden

**Keywords:** Computational modeling, Cell signaling, Network biology, *Rxncon*, Boolean networks

## Abstract

**Background:**

Computational models of cell signaling networks are extremely useful tools for the exploration of underlying system behavior and prediction of response to various perturbations. By representing signaling cascades as executable Boolean networks, the previously developed *rxncon* (“reaction-contingency”) formalism and associated Python package enable accurate and scalable modeling of signal transduction even in large (thousands of components) biological systems. The models are split into reactions, which generate states, and contingencies, that impinge on reactions; this avoids the so-called “combinatorial explosion” of system size. Boolean description of the biological system compensates for the poor availability of kinetic parameters which are necessary for quantitative models. Unfortunately, few tools are available to support *rxncon* model development, especially for large, intricate systems.

**Results:**

We present the *kboolnet* toolkit (https://github.com/Kufalab-UCSD/kboolnet, complete documentation at https://github.com/Kufalab-UCSD/kboolnet/wiki), an R package and a set of scripts that seamlessly integrate with the python-based *rxncon* software and collectively provide a complete workflow for the verification, validation, and visualization of *rxncon* models. The verification script *VerifyModel.R* checks for responsiveness to repeated stimulations as well as consistency of steady state behavior. The validation scripts *TruthTable.R*, *SensitivityAnalysis.R*, and *ScoreNet.R* provide various readouts for the comparison of model predictions to experimental data. In particular, *ScoreNet.R* compares model predictions to a cloud-stored *MIDAS*-format experimental database to provide a numerical score for tracking model accuracy. Finally, the visualization scripts allow for graphical representations of model topology and behavior. The entire *kboolnet* toolkit is cloud-enabled, allowing for easy collaborative development; most scripts also allow for the extraction and analysis of individual user-defined “modules”.

**Conclusion:**

The *kboolnet* toolkit provides a modular, cloud-enabled workflow for the development of *rxncon* models, as well as their verification, validation, and visualization. This will enable the creation of larger, more comprehensive, and more rigorous models of cell signaling using the *rxncon* formalism in the future.

**Supplementary Information:**

The online version contains supplementary material available at 10.1186/s12859-023-05329-6.

## Background

Modeling of cell signaling networks is a crucial tool in the development of the understanding of how said networks operate both normally and pathogenically, providing information which can be used to identify possible therapeutic targets. To this end, a variety of formalisms for describing and simulating these signaling networks have been developed [1–4]. Among these formalisms is *rxncon* (“reaction-contingency”) [4–7], which seeks to overcome two major challenges in cell signaling modeling: the poor availability of kinetic parameters for reactions, and the “combinatorial explosion”, the phenomenon in which the enumeration of all potential states of complexes containing multiple proteins with several possible post-translational modifications results in unwieldy and computationally expensive models [5, 8].

The *rxncon* formalism separates biological signaling networks into two parts: elemental states, which represent information about the status of the system components and reactions, which produce and consume states (see [9] for a detailed explanation of *rxncon’s* model structure and syntax). The bipartite nature of the resulting network solves the problem of the “combinatorial explosion” by eliminating the need for enumeration of all combinations of microstates. The first problem, sparse knowledge of kinetic parameters, is addressed by allowing compilation of *rxncon* models into purely qualitative Boolean networks. Alternatively, *rxncon* models can be compiled into agent- and rule-based models, allowing for pseudo-quantitative simulation of the system [10]; however, compilation into Boolean networks allows for fast and parameter-free evaluation of model dynamics.

In Boolean networks, a system is represented as a set of nodes which can be ON or OFF; the Boolean vector of values of all nodes represents a *state* of the network. The system’s state is updated in discrete synchronous steps where the new value of each node is calculated as a Boolean function of existing values of other nodes. As a result of the finite nature the state space, any simulation of a Boolean network will eventually fall into a finite-size (one or more) loop of Boolean states that the network will indefinitely visit in order; such loop is called an *attractor* and represents a steady state of a Boolean network. Importantly, most Boolean networks have more than one attractor that the system can reach, in a deterministic manner, from different initial states. Due to the enormous size of the state space (2^*n*^ where *n* is the number of states and reactions in a system), the assessment of all possible system trajectories through this space is poorly scalable; however, it can provide great insight into model behavior.

The use of parameter-free Boolean logic and a bipartite network structure makes *rxncon* models efficiently scalable and allows for iterative simulations of extremely large and complex systems (e.g. the previous published *rxncon* model of the cell division cycle of *Saccharomyces cerevisiae* involved 357 unique components, 790 reactions, and 598 contingencies [9]).

Maintaining *rxncon* models of this size presents its own host of challenges. Using currently available software, consistency checks of the model behavior under various combinations of perturbations must be performed manually. A previously developed interface which provided tools for exploration of a model’s topology and Boolean state space is unfortunately no longer available [4]. The published versions of the *rxncon* software provide only limited means for visualizing the model behavior, and no tools to verify it automatically [6]. Furthermore, there is no systematic method of comparing simulation output to pre-existing experimental data or of generating predictions and targets for experimental validation once a final model has been developed. Inspired by *CellNOptR* [11], we present *kboolnet,* a collection of R and Python scripts which serve as a toolkit for the verification, validation, and visualization (VVV) of *rxncon* models. The *kboolnet* toolkit enables separation of full models into smaller, more easily analyzable modules, iterative and collaborative development, and comparison to a manually curated database of responses to various combinations of experimental stimuli and inhibitors.

## Implementation

### Recap of *rxncon*

The *rxncon* (“reaction-contingency”) formalism [4–7] describes a signaling network as a bipartite directed network with nodes of two types: states and reactions. States represent the specific state of a protein at a certain domain or residue level (i.e. what other protein is bound at said domain or a specific covalent modification applied at said residue). Reactions represent uni- or bi-molecular interactions which produce and/or consume states (indicated by directed edges of respective types in the network). States in turn serve as contingencies which either positively or negatively regulate reactions, also indicated by directed edges. By creating a bipartite structure that separates reactions from states, as well as making the states of individual domains/residues on a protein independent of each other, *rxncon* avoids the “combinatorial explosion” often found in rule-based models.

**Fig. 1:**
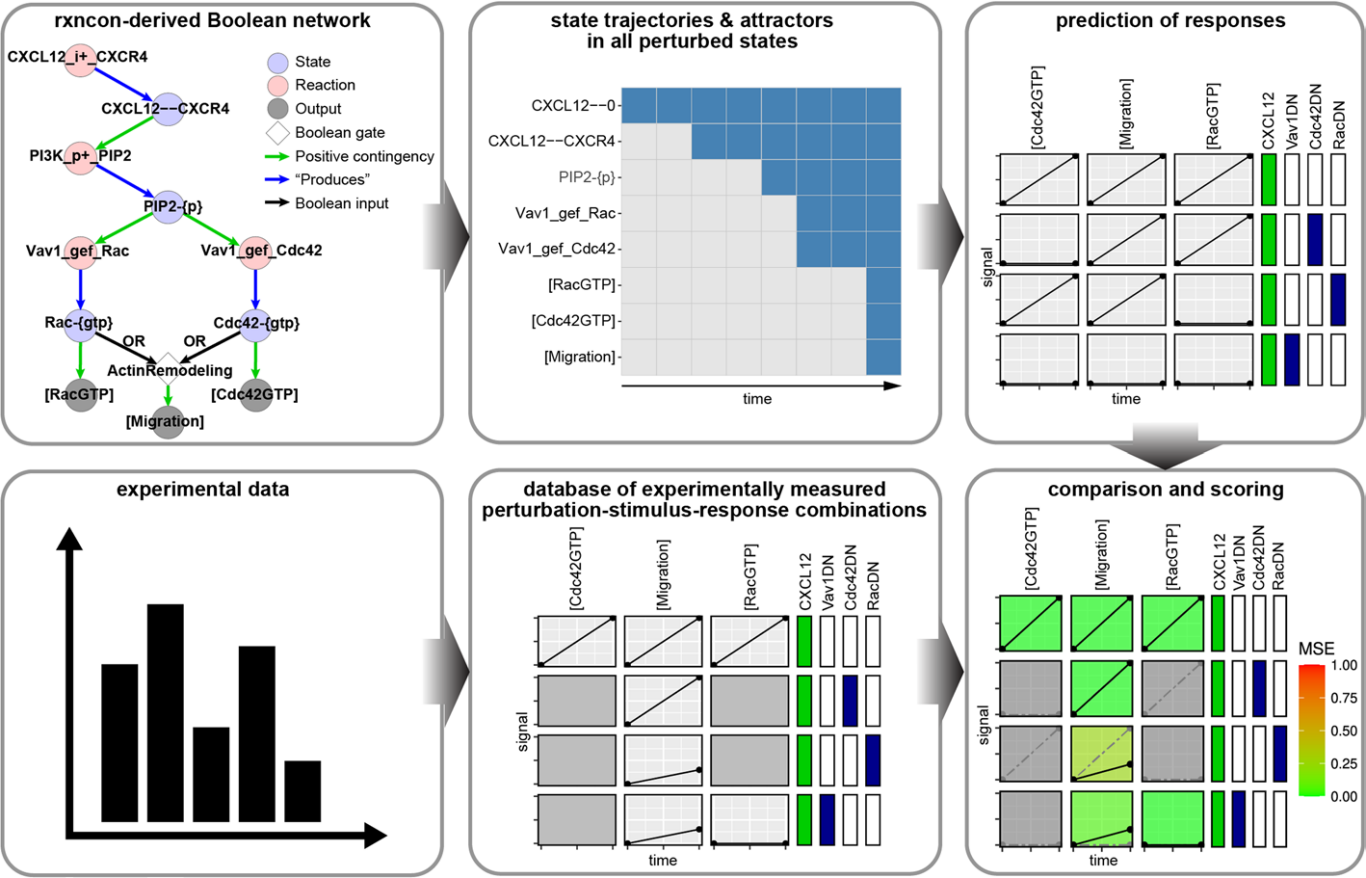
Overall workflow of *ScoreNet.R*. The *ScoreNet.R* script’s workflow takes two main inputs: a *rxncon* model, here pictured by its regulatory graph (reactions [light-red nodes] produce [blue arrows] states [light-blue nodes]; these states serve as positive [green arrow] or negative [red arrows, not shown] regulators of reactions, and as inputs [black arrows] to Boolean gates [white diamonds]) and a database of experimental data. The model is compiled to a Boolean network and simulated under the same stimulus/inhibitor combinations as applied in the experiments in the database. This produces a set of response predictions which is then compared to the experimental database to give a score for the model

The reactions and states are entered, usually semi-manually, in an MS Excel spreadsheet (the primary input format accepted by the *rxncon* software) in accordance with syntactic rules specified in the original publication [5]. Published software [7], written in Python, then parses the spreadsheet and generates either a Boolean model in a format compatible with the Boolean network-based BoolNet simulator [12] or the agent- and rule-based pseudo-quantitative NFsim simulator [10]. Published software [5] also offers several ways of visualizing the network and the resulting simulation trajectories. For example, the so-called *regulatory graph* is a static representation of the bipartite network with nodes and edges color-coded by their types (a small prototype is shown in Fig. 1). Following a Boolean simulation, the trajectory (including the attractor) can be visualized as a matrix where nodes are listed vertically and time ticks horizontally; at each tick, the node can be either on or off as illustrated by the color of the matrix entry (an example in Fig. 1).

### *Kboolnet* implementation overview

*kboolnet* is a system of independent scripts; each script performs a single standalone task and relies on user-provided arguments. Most of the scripts are developed in R (v4.0.2) [13] with RStudio (v1.2.5001) [14], to capitalize on R’s versatile network and data analysis tools. However, the extract_modules.py and reaction_mapping.py scripts are developed in Python (v3.8.3) to provide an interface with the original implementation of *rxncon* [6, 7, 15].

The R scripts require the packages ggplot2 (v3.3.2) [16], dplyr (v1.0.5) [17], openxlsx (v4.2.3) [18], googledrive (v1.0.1) [19], tidyr (v1.1.3) [20], numbers (v0.7.5) [20, 21], xml2 (v1.3.2) [20–22], BoolNet (v2.1.5) [12], egg (v0.4.5) [23], optparse (v1.6.6) [23, 24], igraph (v1.4.2) [25], rappdirs (v0.3.3) [26], and RCy3 (v2.8.0) [27]. The Python scripts require the packages rxncon (v2.0b18) [6, 7, 15], and openpyxl (v3.0.2). Visualizations were generated using Cytoscape (v3.7.1) [28]. The package and its documentation can be found in a GitHub repository located at https://github.com/Kufalab-UCSD/kboolnet/. Installation instructions are located in the repository wiki. Once the *kboolnet* R package is installed, the setupKboolnet() function must be run from an interactive R terminal. Afterward the scripts (and accompanying example files) will be available in a user-determined directory, and can be executed using RStudio’s built-in “sourcing” functionality or from the system command line. A summary of the available scripts can be found in Table 1. Further details of the implementation for *VerifyModel.R*, *TruthTable.R,* and *ScoreNet.R* can be found below.

**Table 1:** Description of *kboolnet* toolkit scripts, their inputs, and their outputs

Type	Name	Description	Inputs	Outputs
Simulation engine	BoolNetSim.R	Used by all the VVV modules below. Simulates a model using the BoolNet package [21] given an initial state and set of nodes to inhibit/activate	Model file, initial state, on-list, off-list	Resulting simulation trajectory and attractor as a CSV and a PDF
Verification	VerifyModel.R	Simulates a model under a set of activators and inhibitors while repeatedly toggling a given ligand on and off to check consistency of the resulting attractors	Model file, set of runs with list including initial state, on-list, off-list, and toggled ligand for each run	RData file holding trajectories and attractors for all rounds of simulation, CSVs and PDFs of consolidated trajectories, and XGMML graphs of the state spaces
Validation	TruthTable.R	Simulates a model under all possible combinations of given inhibitors and activators and sees their effect on a set of output nodes	Model file, nodes to activate or inhibit, output nodes, initial state	Truth table PDF/CSV
	ScoreNet.R	Simulates a model under the perturbations encoded in a database of experimental data and sees how closely the simulation reflects real-world observations	MIDAS-format experimental database, model file	Mean square error for model, PDFs for experimental results, simulation results, and comparison of the two
	SensitivityAnalysis.R	Measures effect of individually inhibiting every node on the effect a ligand has on a set of output nodes	Model file, ligand to toggle, initial state, outputs to monitor	PDF showing semi-quantitative effect of inhibition on ligand to output signal
Visualization	AnimatePath.R	Animates the regulatory graph of a model using a given trajectory CSV	Regulatory graph, trajectory CSV	Animation GIF
	PlotModules.R	Makes individual regulatory graphs of each of modules stored in a model file	Model file, list of modules to plot	Regulatory graph XGMML for each module
	PlotPath.R	Plots a trajectory CSV	Trajectory CSV	Plot as PDF
	PlotPathComparison.R	Aligns two trajectories/attractors and plots an overlay of the two showing (dis)similarities between the two	Two trajectory/attractor CSVs	Comparison plot as PDF

### Evaluation of the attractor space of a *rxncon* model in the presence and absence of stimuli and perturbations: *VerifyModel.R*

The *VerifyModel.R* script enables visualization and exploration of the attractors in the state space that the *rxncon* model visits in response to the addition and removal of ligands, starting from a specified initial state and with an option of forcing selected nodes (states and reactions) to be permanently on or off (to simulate the effect of inhibitors, gene knockdowns, etc.). The script follows a three-step workflow:

***1. Initial simulation round.*** The network is first simulated from the provided initial state, with all ligand nodes off, until reaching an initial “no-ligand” attractor. If no initial state is provided, the default “neutral” state as defined by *rxncon* (all reactions off, all components in their unbound and unmodified states) is used as a starting point. Once the “no-ligand” attractor is reached, its first state is selected, modified such that the ligand is present in its unbound state, and then simulated until reaching an initial “with-ligand” attractor.

***2. Attractor simulation rounds.*** Subsequent “no-ligand” and “with-ligand” attractors are determined as follows: each state within the “with-ligand” attractor (or the only state in the case of a single-state attractor) is modified such that all ligand nodes are off (if the ligand was in a complex with another component, the second component’s unbound state is also turned on to ensure that said component remains within the system). The network is then simulated using these initial states until reaching their corresponding attractors. If these attractors are not identical (which may occur if the “with-ligand” attractor was cyclic), the simulation rounds are stopped and several outputs representing the inconsistent attractors and traversed state space are written to file along with a warning to the user. Attractor identity/similarity is measured using the Hamming distance, accounting for potential phase shifts between the cyclic attractors as described in [29]. If all “no-ligand” attractors reached from the initial states of the “with-ligand” attractor are identical, this process is repeated to determine the next “with-ligand” attractor(s). Each state within the new “no-ligand” attractor is modified such that the ligand is present in its unbound state, and the corresponding “with-ligand” attractors and their similarities are determined.

***3. Meta-attractor determination.*** After each round of attractor simulation, the newly generated “with-ligand” and “no-ligand” attractors are compared to those generated in previous simulation rounds. If both attractors are identical to previously determined attractors, the simulation rounds end as a meta-attractor has been found: this is because the simulation is deterministic. All trajectories and attractors are written to file in both CSV and RData formats for inspection by the user, and a graph representing the trajectory through state space traversed during simulation is written in XGMML format (see *Results* for examples).

### (Pseudo-)quantitative comparison of a *rxncon* model to experimental data: *ScoreNet.R*

*ScoreNet.R* is the heart of the *rxncon* model validation pipeline in *kboolnet*. Its goal is to systematically compare the outputs of the model to experimental data obtained with different sets of stimuli and perturbations. The overall workflow of *ScoreNet.R* is shown in Fig. 1, and the key components described below.

***Cloud-enabled MIDAS-formatted experimental database. ***To facilitate the storage of experimental data in a single cloud-enabled database, we defined a modified version of the MIDAS (Minimum information for data analysis in systems biology) format originally created for *DataRail* [30]. This modified format is machine- and human-readable and allows for the storage of experimental data from heterogeneous sources in a single Excel or Google Sheets file. In our implementation, data is organized into “sub-tables”, each of which describes an experiment, including the treatments applied, data acquisition timepoints, and responses. Mapping of treatments to *rxncon* network nodes is accomplished through a *TreatmentDefs* sheet, which defines the name of a treatment, the effect of the treatment (stimulation, inhibition, or knockout/knockdown), and the *rxncon* nodes affected by the treatment. As the database is intended to include data from various sources, it must be normalized so that data values lie between 0 and 1; such normalization is the responsibility of the user.

***Experimental data pre-processing. ****ScoreNet.R* first merges all “sub-tables” in the provided experimental data file into a single table of all experimental perturbations and the resulting data measurements. To enable comparison with *rxncon* Boolean networks that are not time-resolved and can only predict the (pseudo-)steady state of a system, the merged data must then be collapsed to two timepoints: pre- and post-stimulation. It is assumed that all data values acquired at t = 0 are pre-stimulation. The user can select all other data points (i.e. t > 0) as post-stimulation data points or only data points lying within specific time bins via the script configuration file. After this, all pre-stimulation data values and post-stimulation data values for each output and combinations of stimuli/inhibitor are averaged, resulting in two data points for each output/perturbation combination.

***Generating rxncon predictions and quantifying their agreement with experiments. ***A table of corresponding simulation predictions for each combination of perturbations is then generated as follows: the system is simulated starting from a user-provided initial state (or if not provided, the *rxncon*-generated “neutral” state), with all knocked out and stimulated nodes for the perturbation forced off, until it reaches the “pre-stimulation” attractor. This attractor is then quantified for all outputs by dividing the number of states the output is “on” in the attractor by the total number of states in the attractor, resulting in a value between 0 (always “off”) and 1 (always “on”). An arbitrary state from the pre-stimulation attractor (if the model behavior is internally consistent as determined by *VerifyModel.R*, state choice within the attractor does not affect the outcome) is used as the initial state for another simulation, with all inhibited nodes forced off and all stimulated nodes forced on. The resulting attractor is again quantified and used as the post-stimulation data point. These data points are used to calculate the mean square error (MSE_*p,o*_) for each perturbation combination *p* and output *o*

$${\text{MSE}}_{p,o} = \frac{{\sum\limits_{t = 0}^{1} {\left( {{\text{experiment}}_{p,o} - {\text{simulation}}_{p,o} } \right)^{2} } }}{2}$$

where *t* = 0 is the pre-stimulation time point and *t* = 1 is the post-stimulation time point. The overall mean square error MSE_*tot*_ is calculated as

$${\text{MSE}}_{tot} = \frac{1}{nm}\sum\limits_{p = 1}^{n} {\sum\limits_{o = 1}^{m} {{\text{MSE}}_{p,o} } }$$

where *n* and *m* are the number of perturbation combinations and measured outputs, respectively.

### Dynamic visualization of *rxncon* model trajectories in Boolean state space: *AnimatePath.R*

Animations of the paths generated using *VerifyModel.R* or *BoolNetSim.R* can be made using *AnimatePath.R*. This script functions by automatically creating stills of a regulatory graph of the network (nodes are colored by their ON/OFF status) using the RCy3 [27] API to control Cytoscape [28]. These stills are then joined into a single GIF with numbered frames using the ImageMagick library. An example of such an animation may be found in the Additional File 1.

## Results

### A cloud-enabled *rxncon* modeling workflow

**Fig. 2:**
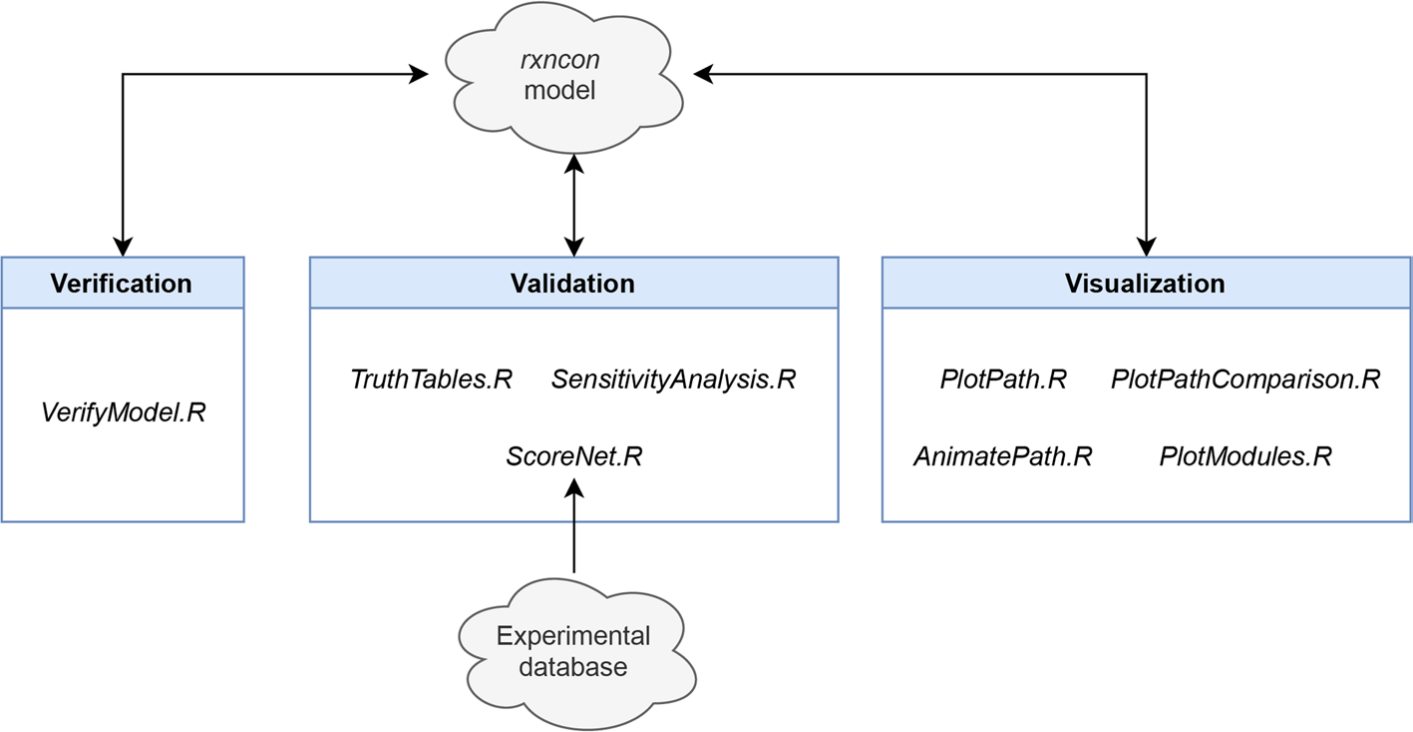
Overall workflow for the *kboolnet* package. A cloud-stored *rxncon* model can be iteratively developed using the three portions of the *kboolnet* workflow: verification of the model’s behavior (responsiveness to ligands and consistency between attractors representing the same biological state), validation against experimental data, and visualization of model trajectories and topology

The *kboolnet* package is designed to provide a variety of verification, validation, and visualization (VVV) tools that together form a cloud-enabled workflow for the development of *rxncon* models (Fig. 2). The workflow extends and automates existing *rxncon* functionality and is centered on analysis of the model once converted to a *BoolNet* Boolean network.

The verification part of the workflow consists of *VerifyModel.R,* a script which can identify and report a variety of model behaviors and responses to stimuli and perturbations. The script performs formal verification of the model by checking the responsiveness of the system to repeated applications of stimuli, outputting the resulting trajectory and a graph visualizing the space of states and attractors reached.

The model validation, which involves the assessment of the model’s agreement with either experimental data or general (experimentally-informed) expectations about the behaviors of its components, is implemented in the form of three scripts: *TruthTable.R*, *SensitivityAnalysis.R*, and *ScoreNet.R*. *TruthTable.R* can be used for quick analysis of the behavior of the model (or portions thereof) by outputting a “truth-table” like graph which shows the response of the system to all possible combinations of a given set of stimuli and inhibitors. *SensitivityAnalysis.R* examines the effect of inhibiting all reactions and knocking out all components individually on a certain set of output nodes under with- and no-ligand conditions. The final script, *ScoreNet.R*, compares behavior of the model to a (modified) *MIDAS*-format experimental database, outputting both visual and numerical metrics that can serve as benchmarks of model quality.

Model visualization is handled by four scripts: *PlotPath.R*, *PlotPathComparison.R*, *PlotModules.R*, and *AnimatePath.R*. *PlotPath.R* outputs a 2D plot of a trajectory where columns represent timepoints and rows represent nodes in the Boolean network, with blue squares indicating “on” states and white squares indicating “off” states. *PlotPathComparison.R* generates a similar visualization after aligning and overlaying two such trajectories, showing their similarities and differences. *PlotModules.R* extracts all user-defined modules from a *rxncon* model file and outputs reaction-contingency graphs for each of them in the XGMML format, allowing for fast visualization of the topology of each module. Finally, *AnimatePath.R* can animate regulatory or state graphs of a *rxncon* network using a trajectory or attractor produced by *VerifyModel.R* or *BoolNetSim.R*. This provides a dynamic representation of the flow of information through the model, potentially providing novel insights about model behavior (an example animation may be found in the Additional File 1).

This workflow is cloud enabled, allowing for the storage of the *rxncon* model and modified *MIDAS*-format experimental data as a Google Sheets file. The above verification and validation scripts, as well as *PlotModules.R*, download the latest version of the model (and experimental database, if necessary). This simplifies iterative and collaborative development of the model.

### Modular tagging and validation of *rxncon* rules

**Fig. 3:**
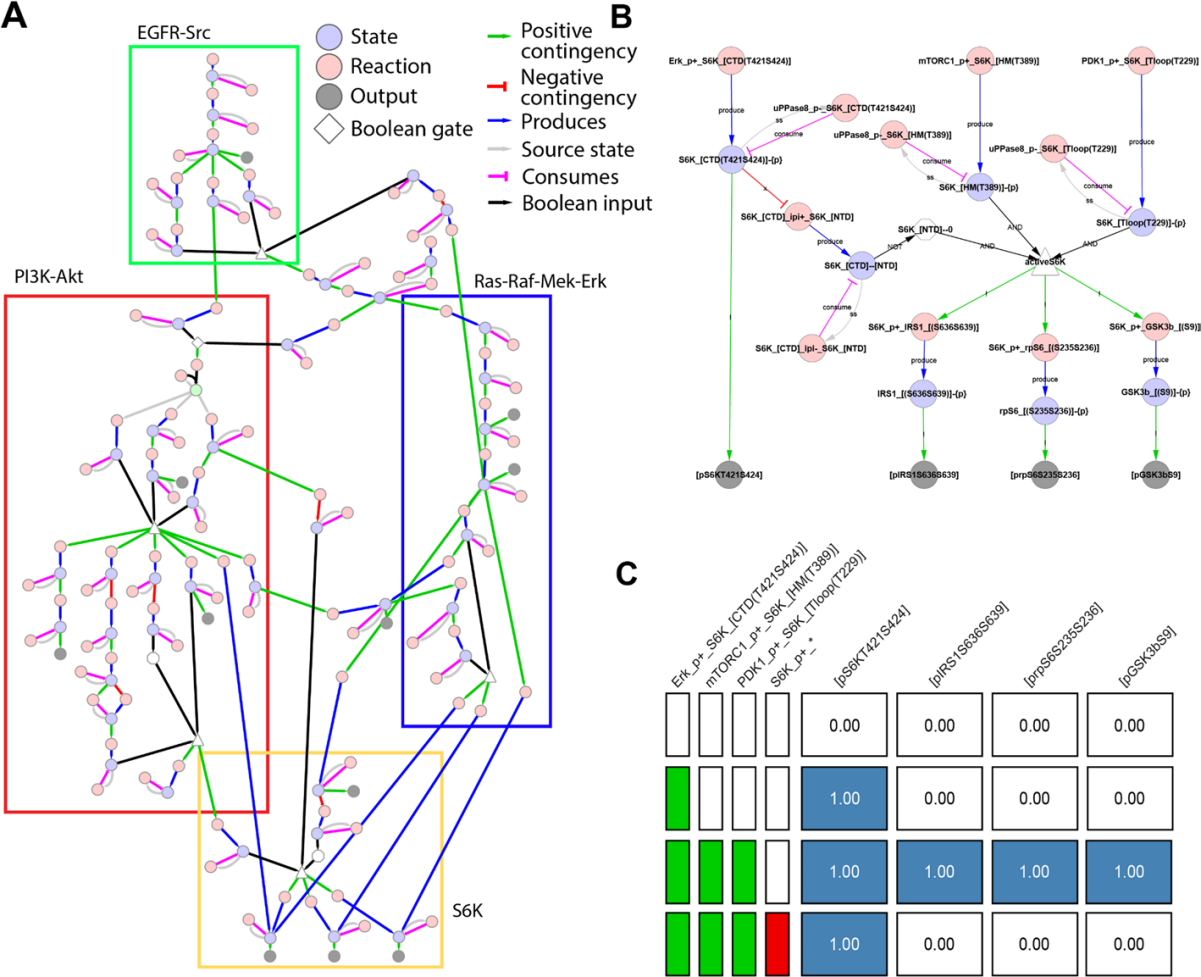
Example of module extraction and qualitative assessment. The full model of EGFR signaling in colorectal cancer cells, a bird’s-eye view of which is presented in (**A**), is broken down into several modules: EGFR-Src (green), PI3K-Akt (red), Ras-Raf-Mek-Erk (blue), and S6K (yellow). Nodes shared between two or more modules are left uncolored. Each of these modules can be extracted for independent visualization and evaluation, as shown for the S6K module in (**B**) and (**C**), using the *PlotModules.R* and *TruthTable.R* scripts, respectively

The model-building process can often result in the definition of large numbers of reaction rules and contingencies (e.g. a model of *Saccharomyces cerevisiae*’s cell cycle included 790 reaction rules and 598 contingencies [9, 30]). This can lead to challenges when attempting to maintain, visualise, and evaluate a large-scale model, prompting us to implement tagging of *rxncon* reactions and contingencies by user-defined module and a numerical quality score. This allows for the extraction of modules for easier analysis as well as inclusion and exclusion of individual reactions/contingencies on the fly (Fig. 3A, B). To implement module tagging, we expanded the format of the original MS Excel (the primary input format for *rxncon* scripts) by adding a new “!Module” column; tagging is implemented using a comma-separated list of module names in this column. Quality tagging exploits the existing “!Quality” column and filters out any reaction or contingency below a given score threshold.

To rapidly and comprehensively characterize the behavior of the extracted modules under various combinations of stimuli and inhibitors, we created the *TruthTable.R* utility. It is inspired by *capso*’s “global truth tables” [31], validation figures presented in previous versions of *rxncon* [6], and truth tables from propositional logic. Given a list of *n* stimuli and inhibitors, the chosen module is simulated for all 2^n^ stimulus/inhibitor combinations beginning from an initial user-provided state (or the *rxncon*-defined “neutral” state). This approach would be impractical for the entire model due to the large number of inputs and output; however, it is very beneficial for the small-size modules. In each simulation, selected stimuli nodes are fixed “on” and all inhibited nodes fixed “off”, the simulation is performed until reaching an attractor, and the output node values are averaged across the attractor states. This results in a score between 0 (always off) and 1 (always on) for each output node; these scores are combined in a matrix-type visualization (Fig. 3C). The output can be used to quickly identify which combinations of stimuli are necessary to activate certain output nodes and which inhibitors abrogate said outputs. The script attempts to “compress” the visualization vertically; that is, if addition of a certain stimulus or inhibitor results in insensitivity of the model to other perturbations, all combinations with said stimulus or inhibitor are combined into a single row. To summarize, *TruthTable.R* facilitates validation of a module against existing knowledge of responses of selected output nodes to combinatorial perturbations.

### Whole-model formal verification

The *VerifyModel.R* script has been designed to facilitate evaluation of dynamic properties of the model as a whole, especially when it is complex and involves elements such as feedback loops. The properties in question are loosely related to those formulated for Petri Nets (a different type of discrete event dynamic systems [32]): reachability and liveliness. Through *VerifyModel.R*, we empirically evaluate attractor reachability for the subgraph of the state graph that is reachable from the given initial state(s) upon the addition and removal of the given set of stimuli or inhibitors.

**Fig. 4:**
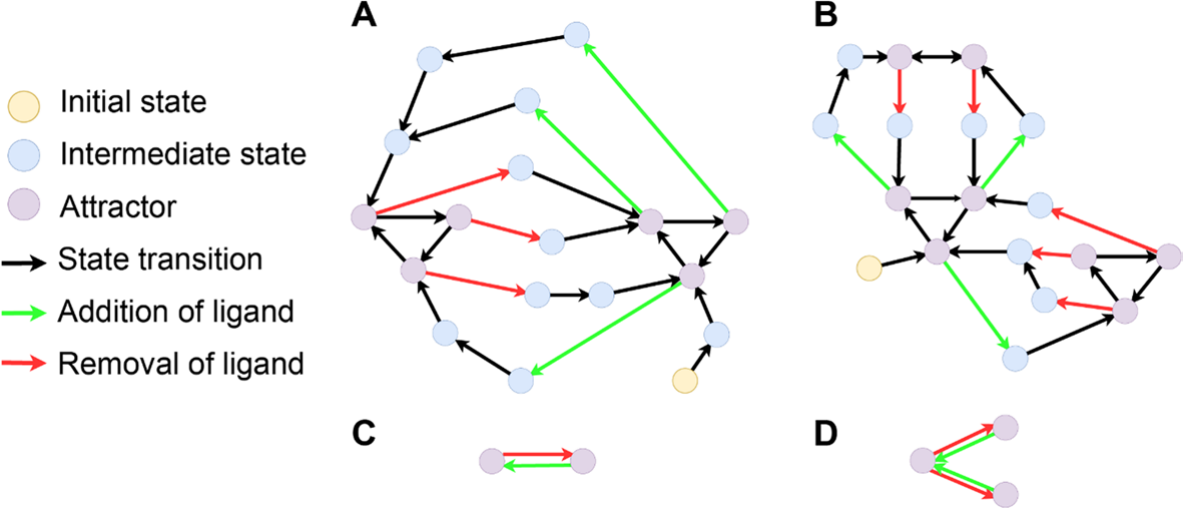
State and attractor spaces for two hypothetical boolean networks. State transition graphs of two *rxncon* networks (**A**, **B**) and their corresponding attractor spaces (**C**, **D**). In (**A**) and (**B**), simulation of the network from intermediate states (blue nodes) according to Boolean state transitions (black edges) causes the system to fall into a cyclic attractor (purple nodes). Addition of the ligand (green edges) or removal of the ligand (red edges) can cause the network to fall into a new attractor. In (**A**) and (**C**), addition of the ligand causes the system to fall into a single “with-ligand” attractor, and removal of the ligand causes the system to return to a single “no-ligand” attractor. This meta-attractor is visualized in the attractor space **C** as a self-consistent and reversible transition between two attractor nodes. In (**B**) and (**D**), removal of the ligand at different timepoints in the “with-ligand” attractor causes the system to fall into two distinct “no-ligand” attractors; this “branching” behavior is visualized in the attractor space (**D)** as a possible transition from a single attractor node to two different (a.k.a. Inconsistent with each other) attractor nodes both corresponding to the same “no-ligand” biological state of the system

We conceptualize a “meta-attractor” as a certain cyclic trajectory through the space of all possible attractors of a Boolean network, with transitions between attractors being achieved by either removal or addition of the ligand (Fig. 4). For many signaling systems (cascades) the addition of the ligand produces changes that are reversible, i.e. the system returns back to its original steady state upon the removal of the ligand. For such systems, the meta-attractor should be simple (Fig. 4A, C): adding the ligand will shift the system into a “with-ligand” attractor, and removing the ligand will shift the system into a “no-ligand” attractor. By contrast, more complex, irreversible behaviors may be encountered e.g. when studying cell division or apoptosis (illustrated by Fig. 4B, D). Finally, some implementations may lead to an unwanted “branching “ behavior where the “with-ligand” attractor is dependent on the specific state within a “no-ligand” attractor at which the ligand is applied, or vice versa. For these scenarios, the “meta-attractor” cannot be defined at all because it is no longer deterministic. In all cases, the ability to detect that a network model becomes “stuck” within a certain attractor or displays a more complex meta-attractor than expected can be helpful in model design and optimization.

**Fig. 5:**
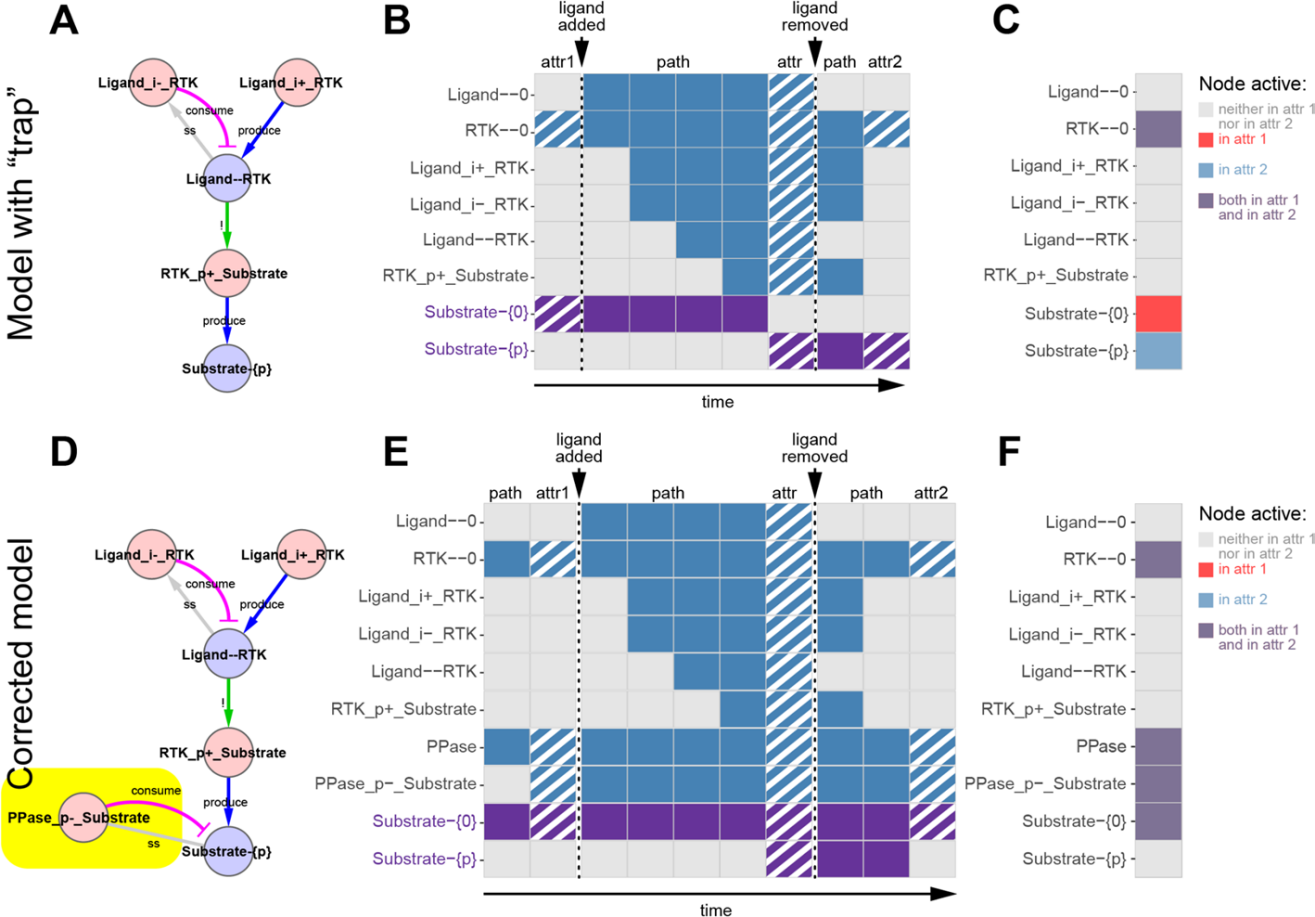
Trap detection with VerifyModel.R. **A** A representative *rxncon* model flagged by *VerifyModel.R* as having inconsistent attractors upon repeated addition and removal of the ligand. **B** This is confirmed by plots of the full paths the network traverses upon the addition and removal of the ligand (attractors are striped, substrate states colored purple). **C** Automatically generated comparison plots reveal that the substrate remains phosphorylated in the first “no-ligand”attractor, but not in the second. **D** This “trap” behaviour is corrected by addition of a dephosphorylation reaction to produce a network (**D**) with consistent no-ligand attractors as shown in (**E**) and (**F**)

Previously, this kind of formal verification had only been possible in *rxncon* by manually adding and removing the ligand in repeated rounds of simulation and comparing the resulting (pseudo-)steady-states/attractors to ensure that the desired system behavior was achieved [15]. Performing verification in this manner is time consuming as it requires repetitive sets of steps (edit initial state, simulate, copy files, edit initial state, etc.). Furthermore, this method of verification only selects a single state within each attractor as the initial state for the next round, leaving open the possibility that undesired “branching” behaviors remain hidden. An improved, automated version of this verification process is available in *VerifyModel.R* (see Fig. 5 for example application, see *Implementation* for more details).

### Model validation through a comparison to database of experimental data

**Fig. 6:**
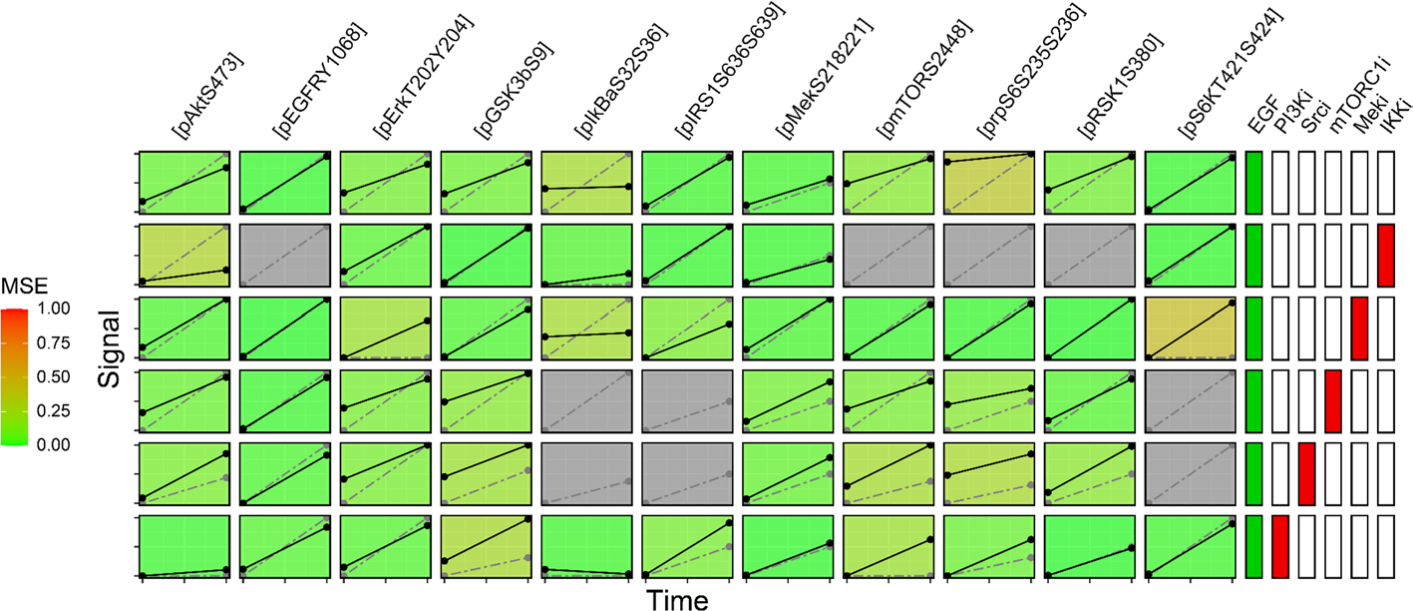
Comparison to experimental data with *ScoreNet.R*. Columns on the left represent output nodes, columns on the right represent combinations of stimuli (green) and inhibitors (red). Cells are colored according to the MSE between experimental data points (black, solid line) and predicted response (grey, dotted line). Cells with no experimental data available are colored grey

Once the model has passed formal verification, it is ready for comparison to experimental data. The *ScoreNet.R* script simulates the network under all combinations of stimuli, inhibitors, and knockouts present in a cloud-stored experimental database. The predicted responses and experimental data are compared and the mean square error (MSE) between the two is calculated (see *Implementation* for details). The calculated MSE ranges from 0 (perfect agreement) to 1 (perfect disagreement) and provides an easily interpretable metric to track progress as a model is iteratively developed. For example, comparing the model presented in Fig. 3A to experimental data collected in SW48 and HT-29 colorectal adenocarcinoma cells [33, 34] resulted in an MSE of 0.092. After the addition of a putative Akt→Cot→RSK edge [35], the MSE dropped to 0.081, a small but measurable improvement (Fig. 6 shows the full output from *ScoreNet.R*).

### A note on performance and scalability

Because *kboolnet* is designed to facilitate the exploration of the overall behavior and state space of a *rxncon* model, its speed and performance are strongly dependent on the size and connectivity of the model. By design, *kboolnet* fully utilizes the performance improvement mechanisms of the *rxncon* formalism (the bipartite graph representation of the network) and its published implementation [6, 7] that compiles models into Boolean networks and takes advantage of the C-accelerated simulation engine of the *BoolNet* R package [12]. Despite these optimizations, we recognize the possibility of decreased scalability when explicitly exploring the state space of the resulting Boolean network, as the number of possible states grows exponentially with the number of nodes. For this reason, *kboolnet* focuses on analyzing only the subset of the model’s state space that is reachable from biologically-relevant initial states. Furthermore, the module tagging, module extraction, and modular validation functionality of *kboolnet* are specifically designed to facilitate the development of large models through a divide-and-conquer approach.

## Conclusion

The *kboolnet* toolkit provides a systematic workflow for the development of *rxncon* models supplemented by a suite of verification, validation, and visualization (VVV) tools. By emphasizing modularity, *kboolnet* simplifies the process of building large and comprehensive models of cell signaling networks spanning multiple biological subunits which can be individually constructed, validated, and visualized. The *kboolnet* suite allows for both qualitative assessments of model integrity and (pseudo-)quantitative comparisons to real biological data. These features are centralized around a cloud-hosted version of both the *rxncon* model and an experimental database, collectively allowing for fast, iterative, and collaborative development of any kind of *rxncon* model. Future development of the *kboolnet* toolkit will expand its capabilities to include pseudo-quantitative modes of simulation supported by the *rxncon* formalism, namely the network-free stochastic simulator *NFsim *[10], as well as expand existing tools for the generation of novel hypotheses and identification of potential drug targets.

## Availability of Data and Materials

The code described and example models analysed in the paper are available on the project GitHub at https://github.com/Kufalab-UCSD/kboolnet/. 

Project name: kboolnet.

Project home page: https://github.com/Kufalab-UCSD/kboolnet/

Operating system(s): Windows, Mac, Linux.

Programming language: R, Python.

Other requirements: See implementation for package dependencies.

License: This software is Copyright © 2023 The Regents of the University of California. All Rights Reserved. Permission to copy, modify, and distribute this software and its documentation for educational, research and non-profit purposes by non-profit organizations, without fee, and without a written agreement is hereby granted, provided that the above copyright notice, this paragraph and the following three paragraphs appear in all copies. Permission for for-profit organizations to make commercial use of this software may be obtained by contacting: Office of Innovation and Commercialization, 9500 Gilman Drive, Mail Code 0910, University of California La Jolla, CA 92093-0910, (858) 534-5815, innovation@ucsd.edu. This software program and documentation are copyrighted by The Regents of the University of California. The software program and documentation are supplied “as is”, without any accompanying services from The Regents. The Regents does not warrant that the operation of the program will be uninterrupted or error-free. The end-user understands that the program was developed for research purposes and is advised not to rely exclusively on the program for any reason. IN NO EVENT SHALL THE UNIVERSITY OF CALIFORNIA BE LIABLE TO ANY PARTY FOR DIRECT, INDIRECT, SPECIAL, INCIDENTAL, OR CONSEQUENTIAL DAMAGES, INCLUDING LOST PROFITS, ARISING OUT OF THE USE OF THIS SOFTWARE AND ITS DOCUMENTATION, EVEN IF THE UNIVERSITY OF CALIFORNIA HAS BEEN ADVISED OF THE POSSIBILITY OF SUCH DAMAGE. THE UNIVERSITY OF CALIFORNIA SPECIFICALLY DISCLAIMS ANY WARRANTIES, INCLUDING, BUT NOT LIMITED TO, THE IMPLIED WARRANTIES OF MERCHANTABILITY AND FITNESS FOR A PARTICULAR PURPOSE. THE SOFTWARE PROVIDED HEREUNDER IS ON AN “AS IS” BASIS, AND THE UNIVERSITY OF CALIFORNIA HAS NO OBLIGATIONS TO PROVIDE MAINTENANCE, SUPPORT, UPDATES, ENHANCEMENTS, OR MODIFICATIONS. Restrictions to use by non-academics: Permission for commercial use by non-profit organizations must be acquired from the UC San Diego Office of Innovation and Commercialization (see license for more information).

## Supplementary Information

**Additional file 1**. An example of an *rxncon* model path animation, generated by the *AnimatePath.R* script.

## Abbreviations

VVV: Verification, validation, and visualization
MIDAS: Minimum information for data analysis in systems biology
MSE: Mean square error
