# Supplementary figures and images for: kboolnet: a toolkit for the verification, validation, and visualization of reaction-contingency (rxncon) models
Source: BMC Bioinformatics. 2023 Jun 12;24:246. doi: 10.1186/s12859-023-05329-6 (PMC10258968; doi:10.1186/s12859-023-05329-6)

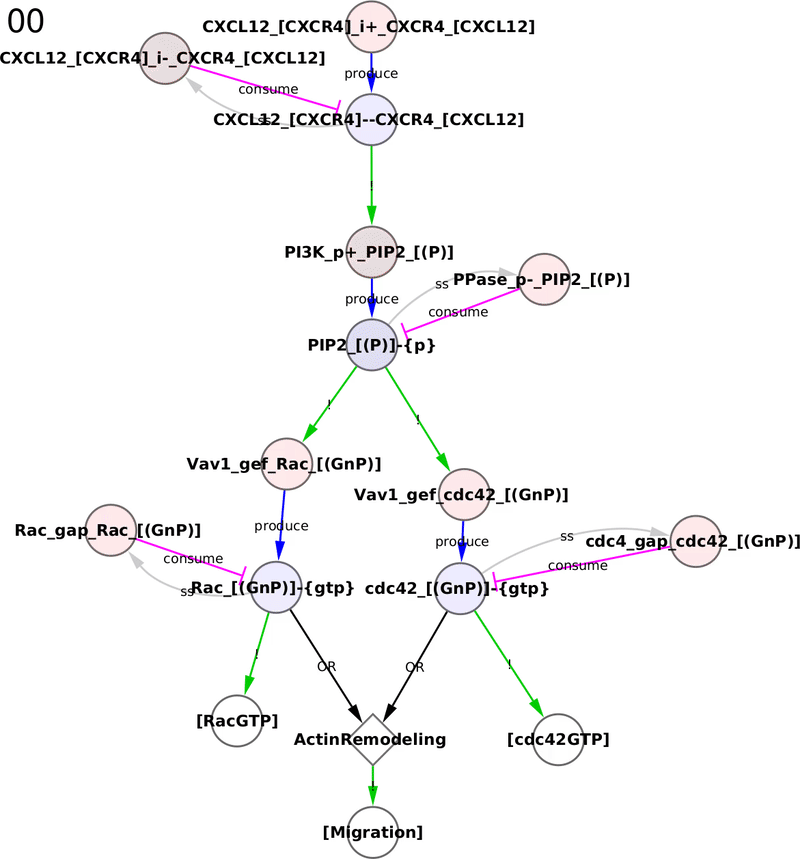

Supplement: Supplementary file 1 — Additional file 1. An example of an rxncon model path animation, generated by the AnimatePath.R script. [file 12859_2023_5329_MOESM1_ESM.gif]
